# Supplementary material for: SUMAMOS EXCELENCIA® Project: Results of the Implementation of Best Practice in a Spanish National Health System (NHS)
Source: Healthcare (Basel). 2021 Mar 28;9(4):374. doi: 10.3390/healthcare9040374 (PMC8066682; doi:10.3390/healthcare9040374)
Supplement: Supplementary file 1 [file healthcare-09-00374-s001.pdf]

RECOMMENDATION FOR ASSESSMENT AND MANAGEMENT OF PAIN (*Registered Nurses' Association of Ontario (2013). Assessment and Management of Pain (3rd ed.). Toronto, ON: Registered Nurses' Association of Ontario)*		***LEVELS OF EVIDENCE***
ASSESMENT		
	Screen for the presence, or risk of, any type of pain:<br>On admission or visit with a health-care professional<br>After a change in medical status<br>Prior to, during and after a procedure	Ib
	Perform a comprehensive pain assessment on persons screened having the presence, or risk of, any type of pain using a systematic approach and appropriate, validated tools	Ib
	Perform a comprehensive pain assessment on persons unable to self-report using a validated tool	III
	Explore the person's beliefs, knowledge and level of understanding about pain and pain management	III
	Document the person's pain characteristics.	IIa
PLANNING		
	Collaborate with the person to identify their goals for pain management and suitable strategies to ensure a comprehensive approach to the plan of care.	Ib
	Establish a comprehensive plan of care that incorporates the goals of the person and the interprofessional team and addresses:<br>Assessment findings<br>The person's beliefs and knowledge and level of understanding<br>The person's attributes and pain characteristics	III

Teach the person, their family and caregivers about the pain management strategies in their plan of care and address known concerns and misbeliefs.	Ib

Levels of Evidence

Ia: Evidence obtained from meta-analysis or systematic reviews of randomized controlled trials
Ib: Evidence obtained from at least one randomized controlled trial.
IIa: Evidence obtained from at least one well-designed controlled studyG without randomization
IIb: Evidence obtained from at least one other type of well-designed quasi-experimental study, without randomization
III: Evidence obtained from well-designed non-experimental descriptive studies, such as comparative studies, correlation studies and case studies.
IV: Evidence obtained from expert opinion or committee reports, and/or clinical experiences of respected authorities.

Adapted from "Annex B: Key to evidence statements and grades of recommendations," by the Scottish Intercollegiate Guidelines Network (SIGN), 2012, in *SIGN 50: A Guideline Developer's Handbook*. Available from http://www.sign. ac.uk/guidelines/fulltext/50/annexb.html

RECOMMENDATION FOR ASSESSMENT AND MANAGEMENT OF THE RISK FALLING *(Registered Nurses' Association of Ontario. (2011). Prevention of Falls and Fall Injuries in the Older Adult)*		***LEVELS OF EVIDENCE***
ASSESMENT		
	Screen all adults to identify those at risk for falls. Conduct screening as part of admission processes, after any significant change in health status, or at least annually.	Ia & V
	Comprehensive assessment (detailed history of falls, routine assessment/history, validated tools, multifactorial assessment as appropriate)	III
PLANNING		
	Implement a combination of interventions tailored to the person and the health-care setting to prevent falls or fall injuries	Ia
	Developed an individualized plan of care in collaboration with the person	Ia

Levels of Evidence

Ia: Evidence obtained from meta-analysis or systematic reviews of randomized controlled trials and/or synthesis of multiple studies primarily of quantitative research.
Ib: Evidence obtained from at least one randomized controlled trial.
IIa: Evidence obtained from at least one well-designed controlled studyG without randomization.
IIb: Evidence obtained from at least one other type of well-designed quasi-experimental study, without randomization.
III: Synthesis of multiple studies primarily of qualitative research
IV: Evidence obtained from well-designed non-experimental observational studies, such as analytical studiesG or descriptive studiesG, and/or qualitative studies.
V: Evidence obtained from expert opinion or committee reports, and/or clinical experiences of respected authorities.

Adapted from the Scottish Intercollegiate Guidelines Network (Scottish Intercollegiate Guidelines Network [SIGN], 2011)

RECOMMENDATION FOR ASSESSMENT AND MANAGEMENT URINARY INCONTINENCE. *(National Guideline Clearinghouse; Agency for Health Care Policy and Research, 2012)*		***LEVELS OF EVIDENCE***
ASSESMENT		
	Document the presence or absence of urinary incontinence (UI) for all patients on admission	I
	For patients with presence of UI, the nurse collaborates with interdisciplinary team members to: Determine whether the UI is transient, established (stress/urge/mixed/overflow/functional), or both and document	I-VI
PLANNING		
	Develop an individualized plan of care using data obtained from the history and physical examination, and in collaboration with other team members. Implement toileting programs as needed	I-VI

Levels of Evidence

I: Systematic reviews (integrative/meta-analyses/clinical practice guidelines based on systematic reviews)
II: Single experimental study (randomized controlled trials [RCTs])
III: Quasi-experimental studies
IV: Non-experimental studies
V: Care report/program evaluation/narrative literature reviews
VI: Opinions of respected authorities/consensus panels

AGREE Next Steps Consortium (2009). Appraisal of guidelines for research & evaluation II. Retrieved from http://www.agreetrust.org/?o=1397

Process and result indicators

**PROCESS INDICATORS. Pain**	
Detection of pain, at admission	Pain detection is defined as identification of pain suffered by the patient (acute, chronic, nociceptive, neuropathic), using a standardised tool.
Detection of pain, after a change in clinical status	Change in clinical status is defined as any significant clinical modification requiring follow-up by a physician.
Pain assessment	Chronic pain assessment is defined as overall pain assessment in persons in whom the presence of pain has been detected, and identification of the type of pain (acute, chronic, nociceptive or neuropathic) using a standardised tool.
Pain management	Establishing and implementing an overall pain management care plan for the patient which would include: evaluation of his/her outcomes, beliefs, knowledge, level of understanding and personal characteristics, and characteristics of the pain.

Patient education, pain management

OUTCOME INDICATORS. Pain

Intensity of pain	Pain assessment using a validated tool, for record-keeping purposes.
Maximum pain	Maximum score or maximum rating reported by a patient after pain-intensity assessment using a validated tool.

PROCESS INDICATORS. Falls

Assessment of risk of falls, at admission or onset of care	Identification of the presence of factors that have been linked in the literature to an increase in falls. A validated tool can be used to classify patients' risk.
Assessment of risk of falls, after a fall	Percentage of patients that have been assessed for risk of falls with a reliable, validated tool, after experiencing a fall.
Prevention of falls	Percentage of patients who are detected to be at risk using a fall-prevention plan or fall-injury reduction programme, based on a multifactorial approach, and are registered.

Use of restraints	Restraints are physical, chemical or environmental measures used to control a person’s physical or behavioural activity or a part of his/her body.<br>Percentage of patients who have physical, chemical or environmental restraints, and are registered.
**OUTCOME INDICATORS. Falls**	
Incidence of falls	Number of falls with or without injury among patients, per 1000 patients/day (acute care/rehabilitation/long stay)<br>Total number of falls with or without injury, per 1000 patients (primary care)
Falls that cause injury	Percentage of falls resulting in mild, moderate, severe injury or death, according to the WHO classification.<br>Injury is defined as bodily harm suffered as a consequence of a fall.
**PROCESS INDICATORS. Urinary incontinence**	
Assessment of presence of urinary incontinence	(Total number of patients in whom the presence of urinary incontinence has been initially assessed, and who are registered / Total number of patients discharged in the assessment period) * 100

Assessment of type of urinary incontinence	(Total number of incontinent patients with identification of type of incontinence, who are registered / Total number of incontinent patients) * 100
Patient education, urinary incontinence management	(Number of patients with incontinence who receive (patients, family members or carers) incontinence management education at least once during the care process, and who are registered / Total number of patients with incontinence)*100
Urinary incontinence management	(Total number of incontinent patients that have a registered, multifactorial urinary incontinence management plan / Total number of incontinent patients) * 100
**OUTCOME INDICATORS. Urinary incontinence**	
Prevalence of urinary incontinence	[Total number of patients with different levels of urinary incontinence 24 hours prior to assessment / Total number of patients attended during the data-collection period] * 100
Impact of urinary incontinence	[Total number of patients with different urinary-incontinence impact levels 24 hours prior to assessment / Total number of patients

	attended during the data-collection period] * 100
Severity of incontinence	Assessment of severity of urinary incontinence with a validated tool.
